# Recent Advancements in Graphene Quantum Dot‐Based Bioimaging and Drug Delivery Systems

**DOI:** 10.1002/mco2.70320

**Published:** 2025-09-23

**Authors:** Sachin Kadian, Shubhangi Shukla, Amit K. Yadav, Brahamdutt Arya, Sushant Sethi, Vishal Chaudhary, Roger Narayan

**Affiliations:** ^1^ Joint Department of Biomedical Engineering University of North Carolina and North Carolina State University Raleigh North Carolina USA; ^2^ Department of Biological Sciences and Engineering Indian Institute of Technology Gandhinagar, Palaj Gandhinagar Gujarat India; ^3^ Department of Higher Education Shiksha Sadan Panchkula Haryana India; ^4^ Mechanical, Materials & Aerospace Engineering Indian Institute of Technology Dharwad Dharwad Karnataka India; ^5^ Physics Department Bhagini Nivedita College, University of Delhi Delhi India

**Keywords:** bioimaging, biomedical applications, drug delivery, graphene quantum dots, heteroatom doping, nanosized graphene

## Abstract

Due to their unique physicochemical, optical, and electronic properties, traditional quantum dots (QDs) have been used for various optoelectronic applications, including semiconductor lasers, photodetectors, transistors, and solar cells. However, unlike other traditional QDs, graphene quantum dots (GQDs), nanosized graphene sheets that possess edge effects and quantum confinement along with a collective structural feature of graphene, have shown less toxicity and desirable biocompatibility, making them an appropriate part of the carbon family for use in biomedical applications. This review article highlights the recent advances and roles of GQDs in healthcare, with a particular focus on their applications involving bioimaging and drug delivery. Furthermore, we provide an overview of the different synthesis methods for GQDs, including the top‐down and bottom‐up approaches, and discuss the modifications that enhance their functionality, such as the incorporation of heteroatoms (e.g., nitrogen, sulfur, and phosphorus) to improve their properties. This review further considers the biological, optical, and toxicological attributes of GQDs, followed by recent developments involving the use of GQDs for drug delivery and bioimaging applications. Last, we describe the challenges, future prospects, and potential directions for advancing the real‐time bioimaging and drug delivery applications of GQDs, including platforms for therapeutic agent release and medical diagnosis.

## Graphical abstract

This article reviews recent advancements and the roles of GQDs in healthcare, with a particular focus on different synthesis methods for GQDs, including top‐down and bottom‐up approaches. It also examines the biological, optical, and toxicological attributes of GQDs, as well as their applications in bioimaging and drug delivery. Additionally, this review discusses the challenges, prospects, and potential directions for translating GQD‐based platforms for real‐time bioimaging and drug delivery applications in clinical settings.

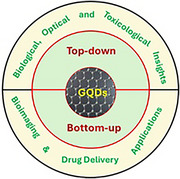

## 1 Introduction

The invention of organic fluorophores and green fluorescence protein has profoundly altered medical research activities in the last few decades [1, 2]. However, the limited photostability of these materials has become a limitation for long‐term bioimaging applications [3, 4]. Semiconductor quantum dots (QDs) have been considered as a promising substitute for organic fluorophores due to their high quantum yield (QY) and exceptional photostability. However, poor water solubility and intrinsic toxicity limit the use of these materials for imaging applications that are related to biomedical research [5, 6, 7, 8]. In addition, the relatively larger size of semiconductor QDs can modify the biological functions of the target molecules [7]. This type of interference can disrupt normal cellular processes, alter protein conformation, or hinder receptor–ligand interactions, potentially compromising the use of the materials for biomedical applications. Furthermore, concerns regarding the toxicity of heavy metals commonly used in traditional QDs, such as cadmium and lead, have raised biocompatibility issues. The shortcomings associated with organic and inorganic QD‐based fluorescent probes led to several efforts to investigate novel fluorescent materials for biomedical applications, including bioimaging and drug delivery [7].

As a result, a nanoscopic material of the carbon family, namely, graphene QDs (GQDs), has attracted considerable attention due to its unusual physical properties, chemical properties, optical properties, and desirable biocompatibility [3, 9, 10]. In addition, GQDs show several attractive characteristics, such as excellent aqueous solubility, high fluorescence QY, tunable bandgap, good photostability, and the presence of numerous active functional groups, including carboxyl, carbonyl, hydroxyl, and epoxide groups [11, 12, 13, 14]. Due to these unusual characteristics, GQDs can network with different kinds of biological molecules and other substrates through these active functional groups via π–π, covalent, physicochemical, and electrostatic interactions, offering several approaches for the development of nanodevices for bioimaging, disease diagnosis, and drug delivery applications [15, 16]. In addition to their functional, structural, chemical, and biological characteristics, GQDs also exhibit intrinsic photoluminescence (PL), making them useful for optical biosensor and bioimaging applications. Therefore, GQDs are being actively explored for their ability to overcome the limitations of conventional probes while providing multiple characteristics that are suitable for a broad range of biomedical applications. Initially, Pan et al. [17] described the hydrothermal synthesis of blue fluorescent GQDs by slicing large graphene sheets and demonstrating the fluorescent properties of these materials. Several research groups subsequently reported their efforts to explore the potential bioimaging and drug delivery applications of the materials [17].

Although GQDs possess several attractive properties, their relatively low QY and brief fluorescence lifetime impede their use for commercial biomedical applications. In order to overcome these concerns, surface functionalization and heteroatom doping have been considered as suitable approaches to optimize the optical and biological characteristics of GQDs [9, 18, 19, 20, 21]. These alterations in the biological, physical, chemical, and optical properties of GQDs can be achieved after as well as during the preparation of GQDs. Several top‐down and bottom‐up synthesis methods, including hydrothermal treatment, electrochemical treatment, physical grinding, microwave treatment, thermal pyrolysis, and incomplete carbonization, have been utilized for optimizing the structure, size, shape, and other characteristics of GQDs [22, 23, 24, 25]. For example, various heteroatom dopants, such as boron (B), nitrogen (N), phosphorus (P), fluorine (F), sulfur (S), chlorine (Cl), selenium (Se), and silicon (Si), have been noted to be useful for modifying physical, chemical, and optical properties of GQDs [13, 26, 27, 28, 29, 30, 31, 32, 33]. Since the optimum wavelength light can be exploited through heteroatom doping, doped GQDs with tunable fluorescence have the capability to be employed in bio‐imaging applications and bio‐sensing applications. Furthermore, the active hydroxyl and carboxyl groups on the GQD surface as well as on the GQD edges can provide desirable hydrophilicity, which can enhance biocompatibility and stability [25, 26, 33]. In addition, the presence of these surface and edge functional groups allows for the straightforward loading of various biomolecules, such as chemotherapeutic agents and cancer cell targeting receptors, which can be utilized for targeted drug delivery and bioimaging applications [34, 35].

**FIGURE 1:**
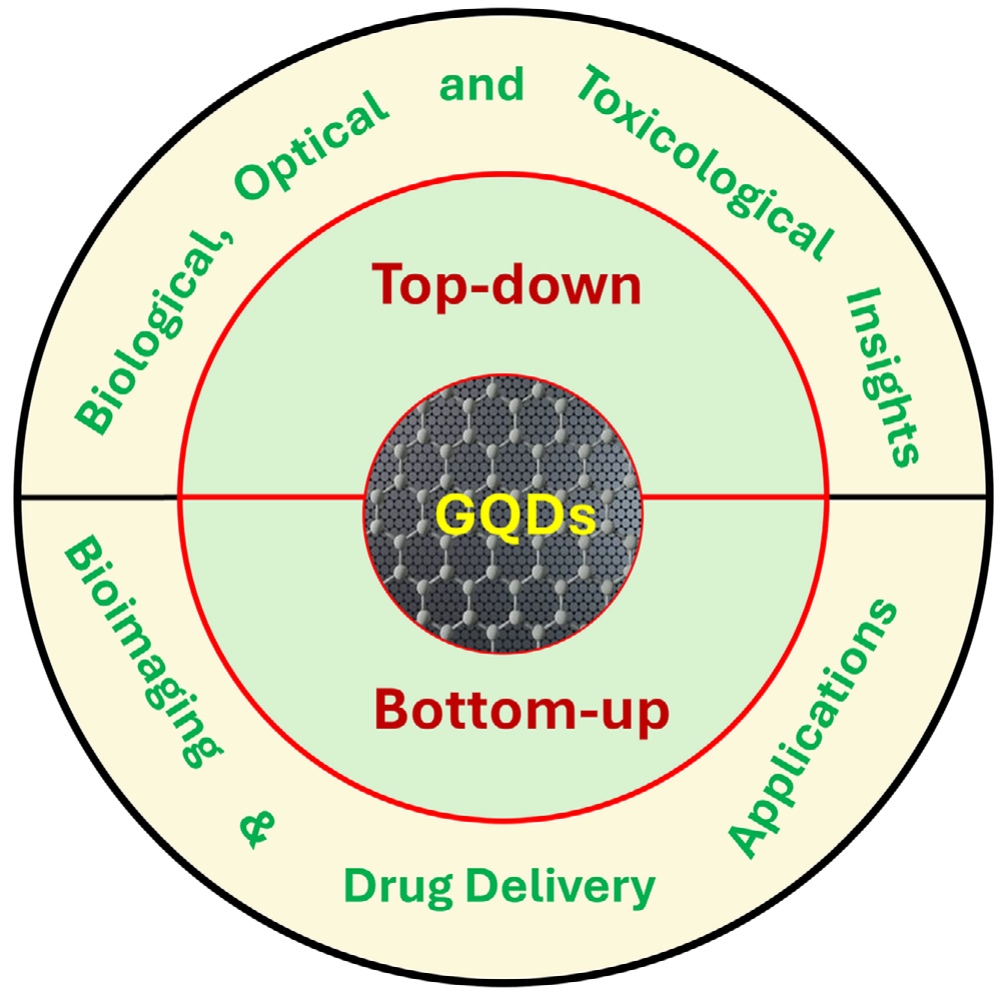
Schematic overview of the review article structure, highlighting recent advances in synthesis strategies (top‐down and bottom‐up approaches) of GQDs, their biological, optical, and toxicological attributes, as well as applications in bioimaging and drug delivery systems.

In contrast to traditional core–shell nanoparticle‐based drug delivery systems, in which the wrapping of the core limits the available drug loading sites, these GQDs offer free π‐orbitals and high surface area for loading of the drug molecules via π‐stacking, which can enable the creation of a targeted drug delivery system [34, 35, 36, 37, 38]. Due to these advantageous characteristics, several research groups evaluated the synthesis of GQDs via various easily accessible precursors with enhanced fluorescence, biocompatibility, aqueous solubility, and drug loading capacity. However, several technological gaps remain, such as scalable manufacturing for mass production. This review article considers the latest advances in GQDs research, with a focus on bioimaging applications and drug delivery applications, as shown in Figure 1. The first section highlights various common and efficient bottom‐up and top‐down synthesis processes to prepare heteroatom‐doped and undoped GQDs, as well as their advantages and disadvantages. The next section provides information on the biological, optical, and toxicological attributes of GQDs and their applications in bioimaging and drug delivery systems. The last section describes current issues and future directions related to the real‐time bioimaging and drug delivery applications of GQDs.

## 2 Synthesis Approaches of GQDs

The synthesis methods of GQDs generally depend on either the breakdown of the macroscopic/bulk precursor material into a nanoscale size through various physical, electrochemical oxidation, or chemical ablation methods (belonging to the top‐down processing approach category) or the controlled chemical fusion of the atomic precursor molecules via pyrolysis or carbonization (belonging to the bottom‐up processing approach category) [12, 39, 40, 41]. Many of the synthesis approaches belonging to these two groups are straightforward and economical; these approaches utilize easily accessible raw materials such as graphene, carbon black, carbon nanotubes, fullerenes, graphite sheets, graphene oxide (GO), citric acid, urea, glucose, as well as other aromatic, conjugated, and nonconjugated compounds [42]. Several research reports have mentioned that both the synthesis method and the precursor materials play a key role in the chemical, physical, optical, and biological properties of GQDs. The synthesis via top‐down methods is typically associated with low production yields, chemical hazards, and long reaction times. In contrast, bottom‐up methods offer comparatively higher production yields, utilize economic organic precursors, require shorter reaction times, and provide convenient heteroatom doping during the synthesis process [43]. The next subsection describes recent progress in synthesis techniques associated with these two processing approach categories.

### 2.1 Top‐Down Approaches

**FIGURE 2:**
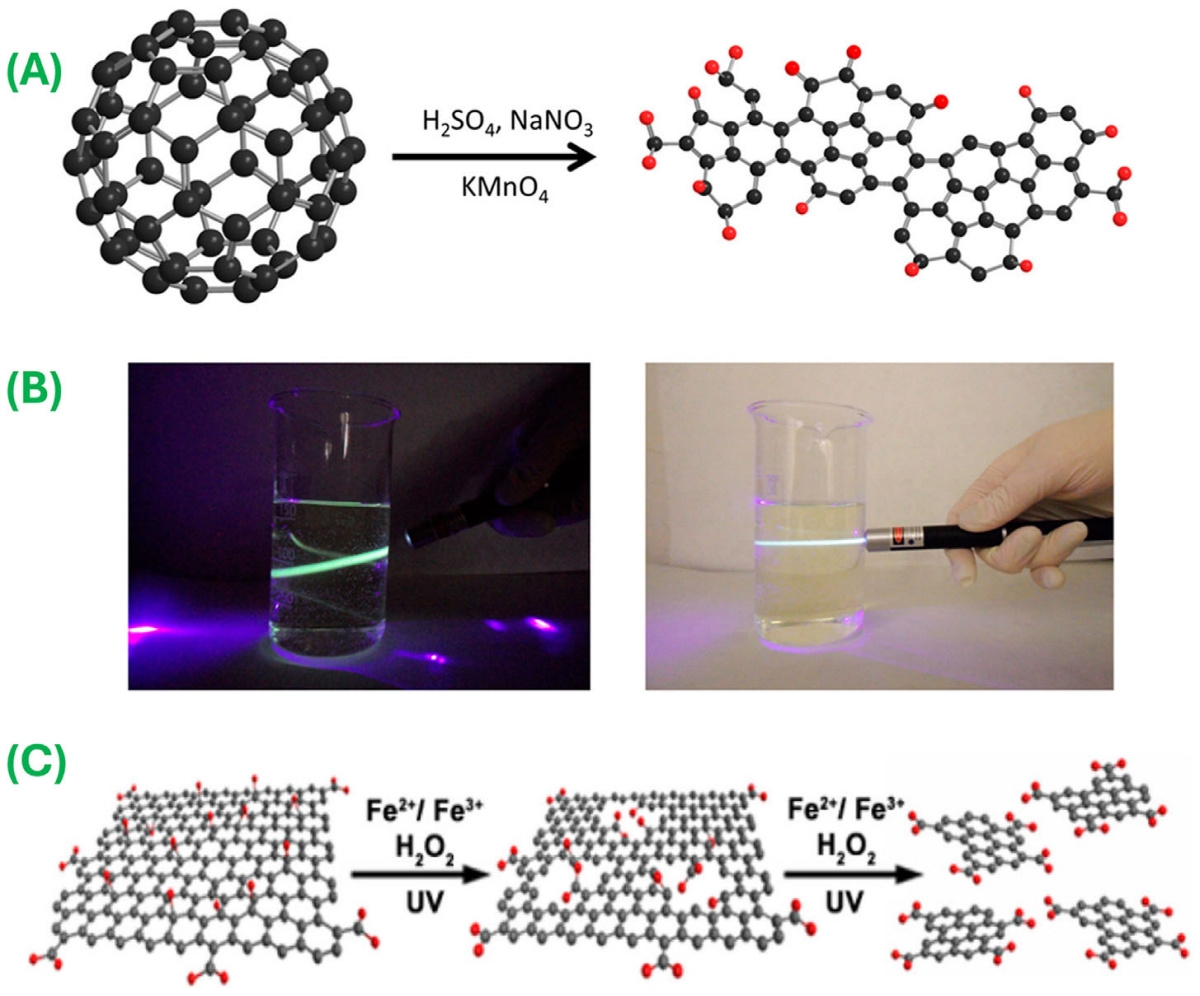
Top‐down synthesis approaches. (A) Synthesis of GQDs through oxidation and cage‐opening of fullerene C_60_ in the presence of chemical oxidant and strong acid, and (B) illustration of luminescence of GQDs under irradiation from a 405 nm blue laser pointer. (Reproduced with permission from Ref. [48], Copyright 2015 American Chemical Society.) (C) Synthesis of GQDs via photo‐Fenton reaction of graphene oxide. (Reproduced with permission from Ref. [56], Copyright 2015 American Chemical Society.)

The top‐down processing approach involves breaking down the bulk carbonaceous precursor materials, including carbon fiber [41, 44], graphite [45, 46], carbon black [47], fullerenes [48], GO [49], graphene [50], and carbon nanotubes [51] into nano‐size graphene sheets via various processes, including electrochemical oxidation, physical grinding, chemical ablation, and ion beam treatment. For example, Luo et al. [52] considered processing white fluorescent GQDs by cleaving large graphite sheets via sonication in a strongly acidic medium, followed by microwave treatment for 12 h. The obtained white fluorescent GQDs demonstrated excellent optical and electronic properties and were employed for the preparation of a white light‐emitting diode. Next, Ye et al. [53] prepared GQDs using coal as a precursor through a chemical oxidation process. They concluded that it is straightforward to cleave the weakly bonded nano‐sized graphitic component of coal through the oxidation process [53]. Chua et al. [48] demonstrated the synthesis of 2–3 nm size GQDs via cage‐opening (Figure 2A) and fragmentation processes of fullerene using a mixture of a strong chemical oxidant and acid. The as‐synthesized GQDs showed bright luminescence properties at 460 nm under a 340 nm excitation wavelength (Figure 2B) and persisted in a completely dispersed state in an aqueous solution. Similarly, Dong et al. [47] used CX‐72 carbon black as a precursor for synthesizing single‐ and multilayered GQDs via a chemical oxidation process [47]. Both the single‐ and multilayered GQDs demonstrated excellent optical, biological, chemical, and electronic properties for the development of bioimaging and optoelectronics devices, respectively. Although the raw materials utilized in these processes are straightforward to obtain, the disposal process for the hazardous oxidants can be an expensive activity. Zhu et al. [54] demonstrated a straightforward acid and by‐product‐free synthesis of GQDs by oxidizing GO through hydroxyl radicals (˙OH) achieved from the catalytic decomposition of H_2_O_2_. They mentioned that the resultant GQDs can be utilized without modification for in vitro fluorescence imaging. Similarly, Liu et al. [55] reported acid‐free production of GQDs through the oxidation of coal tar pitch using hydrogen peroxide under a mild environment. The as‐prepared GQDs were highly fluorescent and monodisperse with a size of 1.7 ± 0.4 nm, which corresponded to a few layers of graphene. Consequently, several attempts have been made to synthesize shape and size‐controlled GQDs using different raw materials. For instance, Zhou et al. [56] described the synthesis of carboxylic group‐enriched GQDs by cleaving the C─C bonds of oxygen‐containing groups (Figure 2C) in the GO sheet through a photo‐Fenton reaction process under UV radiation. They mentioned that the reaction rate was dependent on the oxidation degree of the GO; the as‐prepared GQDs demonstrated the ability to be employed in various biomedical applications. Huang et al. [57] used an ammonia solution as an electrolyte to accelerate the electrochemical oxidation and cutting of graphene paper to obtain a high yield of GQDs. The results indicated that the yield of as‐prepared GQDs was 28 times higher than that for GQDs prepared via a strong electrolyte‐based synthesis process. Moreover, a change in the concentration of electrolytes can result in amino‐functionalized GQDs, which can be used for several biomedical applications. Similar to the electrochemical oxidation and cutting of carbonic precursors, reducing compounds have also been applied to cleave the C─O bond within oxidized carbonaceous materials. For instance, Rajender and Giri [58] used GO as a precursor for synthesizing GQDs through a hydrothermal process. They observed that during the hydrothermal reaction, the epoxy groups connected at the defective sites cleave the GO sheets into GQDs. Similarly, Tayyebi et al. [59] used supercritical water for the synthesis of size‐tunable GQDs via the instantaneous destruction and reduction of oxidized graphene sheets. The thermal conductivity results for these materials revealed the potential of as‐prepared GQDs in photothermal therapeutic applications. Tetsuka et al. [60] synthesized highly fluorescent specific edges and amino‐functionalized GQDs from GO pieces. They mentioned that the optical properties of these GQDs can be optimized through selective edge functionalization. However, many limitations, including low production yields, complex synthesis processes, expensive instruments, expensive raw materials, hazardous chemicals, hazardous reagents, and extreme conditions, impede mass production and commercialization via these approaches.

### 2.2 Bottom‐Up Approaches

**FIGURE 3:**
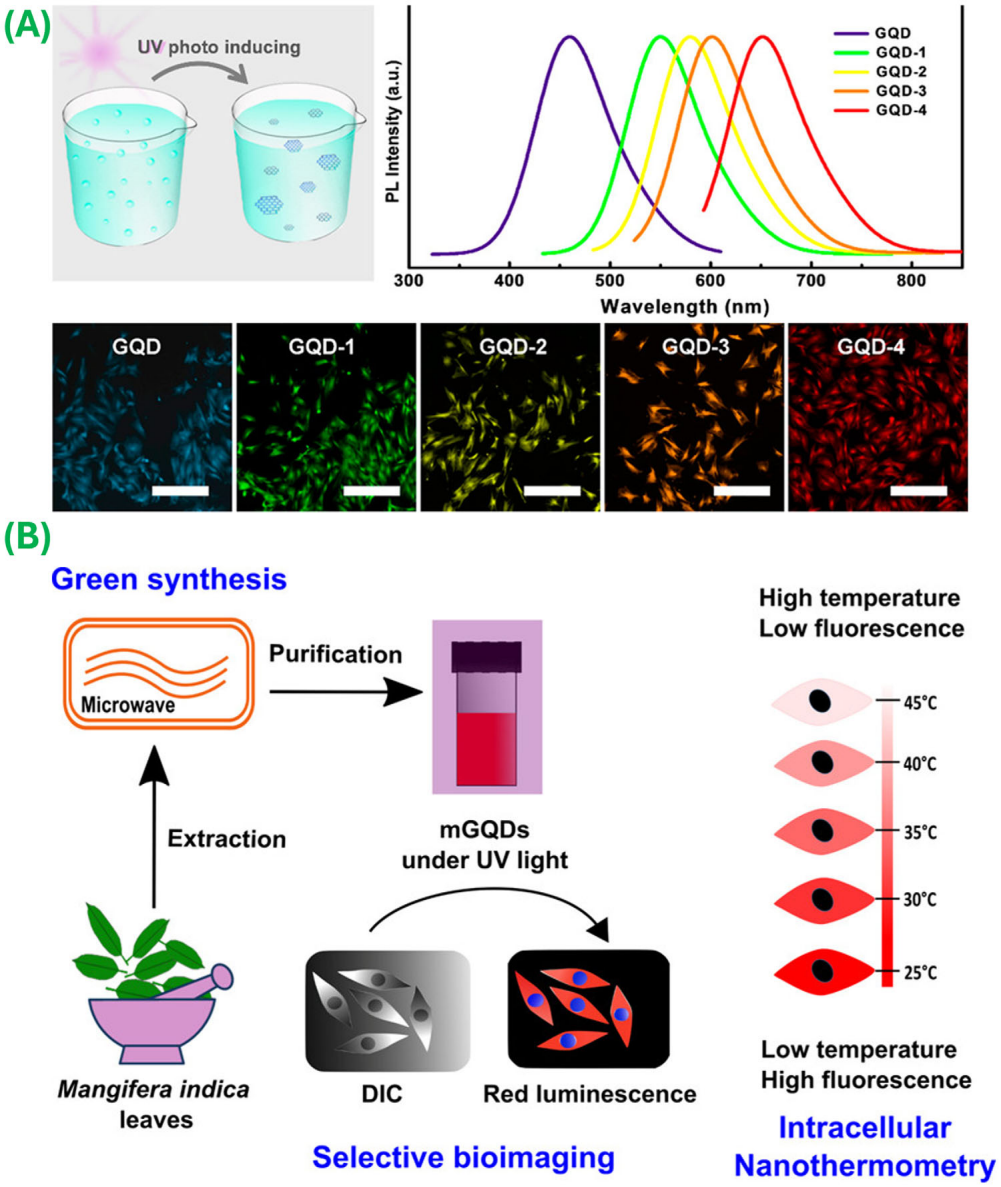
Bottom‐up synthesis approaches. (A) Preparation of GQDs through free‐radical polymerization of oxygen‐containing aromatic compounds under ultraviolet irradiation (reproduced with permission from Ref. [66], Copyright 2017 American Chemical Society), and (B) microwave‐assisted green‐synthesis of red‐luminescent GQDs via ethanolic extracts of Mangifera indica (mango) leaves. (Reproduced with permission from Ref. [67], Copyright 2017 American Chemical Society.)

The bottom‐up processing approach involves the controlled chemical fusion of the atomic precursor molecules, including glucose, amino acids, folic acid, aspartic acid, and citric acid, through cage opening, thermal pyrolysis, incomplete carbonization, hydrothermal, and microwave processes. For example, Dong et al. [25] prepared 15 nm wide and 0.5–2.0 nm thick GQD sheets through incomplete carbonization of citric acid and mixing the resultant liquid into alkaline solutions. The obtained GQDs exhibited an excitation‐independent fluorescence emission and a relatively high QY. Lu et al. [61] demonstrated the fragmentation of C_60_ molecules at a high temperature to generate carbon clusters, which further diffuse and aggregate to obtain the GQDs. The structure of the materials, which was visualized via scanning tunneling microscopy and confirmed by DFT calculations, indicated that the GQDs were formed via the cage opening of C_60_. Similarly, Chua et al. [48] used fullerene as a precursor material for the preparation of 2–3 nm size GQDs by treating it with strong oxidants to initiate the fragmentation process and cage‐opening of fullerene C_60_. The obtained GQDs demonstrated strong fluorescence under 340 nm light with emission at 460 nm; the authors reported that the optical characteristics can be modified by altering the oxidizing agents. Zhang et al. [62] used the mixture of aspartic acid and NH_4_HCO_3_ as a raw material to synthesize blue fluorescent GQDs via a one‐step microwave‐assisted pyrolysis method. The synthesized GQDs exhibited a high QY and were demonstrated for use in bio‐imaging applications. Jeon et al. [63] described a novel method for preparing N‐, S‐codoped GQDs, and N‐doped GQDs (N‐GQDs) via a solvothermal process involving norepinephrine and changing the solvents under microwave irradiation. The 3–4 nm GQDs obtained from the reported solvothermal process exhibited outstanding photocatalytic activity. Kadian et al. [9] prepared highly fluorescent S‐doped GQDs (SGQDs) through the pyrolysis of 3‐mercaptopropionic acid and citric acid. The obtained SGQDs showed exceptional hydrophilicity, high fluorescence, and higher QY when compared with the undoped GQDs; the authors further modified the surface of these SGQDs with folic acid and used the materials for targeted imaging of cancer cells [15, 64]. Khodadadei et al. [65] utilized citric acid and urea as precursor materials for synthesizing blue‐fluorescent N‐GQDs via a hydrothermal process; they loaded the N‐GQDs with the drug methotrexate for use as a nanocarrier and drug delivery system. The hydrothermal, solvothermal, carbonization, and pyrolysis approaches discussed above commonly require hot air ovens, heating mantles, and hot plates; the use of these tools leads to some temperature variation inside the reactor, which creates inconsistencies in the structure, shape, and size of the final product. Due to the even distribution of heat inside the reactor, a microwave‐aided bottom‐up method has been introduced as an alternative to conventional approaches, which leads to the uniform production of GQDs. For example, Zhu et al. [66] used a microwave oven to synthesize water‐soluble ultraviolet photoluminescent GQDs (Figure 3A) by heating a glucose solution in the presence of ammonia. They mentioned that by changing the reaction time and microwave power, they were able to alter the optical properties of the GQDs. Similarly, Kumawat et al. [67] utilized an ethanolic extract that was made using Mangifera indica leaves (Figure 3B) as a raw material to prepare uniformly sized red fluorescent GQDs via a microwave‐assisted approach. These GQDs exhibited excellent optical properties, biocompatibility, and cellular uptake by L929 cells; the materials were employed for bioimaging applications. Further, Hasan et al. [68] developed N‐GQDs as well as N‐ and S‐codoped GQDs through the single‐step microwave treatment of glucosamine–HCl and thiourea precursors, respectively. The as‐synthesized, uniform‐sized, well‐dispersed GQDs demonstrated bright fluorescence in the NIR and visible range with a high QY.

Both bottom‐up as well as top‐down methods have been widely utilized for synthesizing doped and undoped GQDs. These synthesis strategies are associated with several disadvantages and advantages, as summarized in Table 1. The main benefits of the top‐down methods are the adequate availability of the precursors, economics of scale, and ease of the procedure; the utilization of hazardous acids, prolonged synthesis times, and poor production yields limit the use of top‐down methods in terms of mass manufacturing of GQDs for commercialization. Compared with other methods, hydrothermal and solvothermal approaches have been extensively evaluated among the numerous top‐down methods. Further, the bottom‐up methods require various carbonaceous molecules, including glucose, amino acids, folic acid, aspartic acid, and citric acid. However, bottom‐up methods are effective in producing doped, tailored, functionalized, and size‐controlled GQDs in a few minutes. Out of these bottom‐up methods, microwave‐aided production is described as a rapid, straightforward, and environmentally responsive approach for GQD synthesis.

**TABLE 1:** Properties, advantages, and disadvantages associated with the top‐down and bottom‐up synthesis methods for preparing GQDs.

Methods	Subcategory	Precursors	Lateral size (nm)	PLQY (%)	Advantages	Disadvantages	References
Top‐down	Electrochemical	Graphite Graphene	5–10 3–5	14 –	Facile method and high fluorescence quantum yield	Multiple types precursors required	[69] [70]
	Hydrothermal	Graphene sheets GO sheets	5–13 5–10	6.9 –	Narrow size distribution	Prolonged and acidic oxidation process	[17] [71]
	Solvothermal	GO	3–5	9.9	Narrow size distribution	Time‐consuming process	[72]
	Microwave‐assisted hydrothermal	Glucosamine	6	32	Improved fluorescence quantum yield, shortened reaction time, and narrow size distribution	Sophisticated equipment	[73]
	Acidic oxidation	GO Coal CX‐72	13.3 2.96 15	7.4 51 4.04	Low‐cost raw materials	Wide size distribution, difficult to eliminate the acid oxidizing agent	[74] [53] [75]
	Ultrasonic shearing	GO sheets	7.31	33	Improved fluorescence quantum yield and narrow size distribution	Lengthy process to obtain precursors	[76]
Bottom‐up	Hydrothermal	CA + diethylenetriamine CA + ethylenediamine	∼3 5–10	76.8 75.2	Low‐cost and environmentally friendly approach, high fluorescence quantum yield, easy to dope, size control, and shortened reaction time	Limited carbon‐containing raw materials and precursors, such as aromatic structural molecules	[10] [77]
	Microwave‐assisted	Glucosamine–HCl and thiourea	5.50	60			[68]
	Step‐wise organic synthesis	Salicylic acid	1–5	86			[66]
	Soft‐template	CA + ethylenediamine	2	83			[78]
	Intermolecular condensation	CA + l‐cystine	2.8	84			[79]
	Pyrolysis or carbonization	CA CA+MPA	∼15 ∼4	9 57			[25] [9]

Abbreviations: GO: graphene oxide, PLQY: photoluminescence quantum yield, CA: citric acid, HCl: hydrochloric acid, MPA: mercaptopropionic acid.

## 3 Biological, Optical, and Toxicological Insights into GQDs

With the development of nanoscale processing and characterization methods, the biomedical applications of engineered nanomaterials have grown significantly. This development has attracted increased attention toward the potential health and environmental impacts of GQDs, particularly when these nanomaterials are being used in living systems [80]. Consequently, toxicology approaches have been used to systematically study the toxicity, safety, optical characteristics, and biological characteristics of these newly developed nanomaterials. Among these various nanomaterials, carbon‐based structures like GQDs have attracted great interest due to their unique optical properties, small size, and surface tunability [81]. In particular, GQDs have shown excellent potential for applications such as bioimaging, drug delivery, and diagnostics due to their intrinsic biocompatibility and PL. However, to realize their full clinical potential, a thorough understanding of the biosafety and nanotoxicological profile of GQDs is necessary. Assessing the influences of GQDs at the cellular and systemic levels is very important to ensure safe and effective use of these materials in medical settings [82]. In this section, we have highlighted the optical properties, biological properties, biosafety, toxicity, and nanotoxicology characteristics of GQDs.

### 3.1 Biological Attributes of GQDs

GQDs have attracted interest because of their excellent biocompatibility and aqueous solubility. In contrast to pristine graphene, GQDs contain various oxygen‐rich functional groups on their edges and surfaces, which enhance their dispersibility in water. Due to the presence of carbon in their structure, GQDs exhibit low inherent toxicity and rarely accumulate in tissues, in contrast to metal nanoparticles. However, the incidence of certain functional groups in GQDs can alter the toxicity levels [83]. Some studies have confirmed the general biosafety of GQDs; however, surface modifications can lead to variations in cytotoxic responses [84]. For instance, GQDs functionalized with hydroxyl groups tend to exhibit increased cytotoxicity at concentrations above 100 µg mL^−1^; in contrast, GQDs having carboxyl (‐COOH), dimethylamino (‐CHON(CH_3_)_2_), and amine (‐NH_2_) groups show minimal toxicity even at concentrations up to 200 µg mL^−1^ [85]. Further, under light exposure, GQDs can generate singlet oxygen and other reactive oxygen species (ROS), which may cause oxidative stress in biological systems. Photo‐induced generation of ROS, while a concern for cytotoxicity, can be advantageous in therapeutic contexts such as photodynamic therapy (PDT) [86]. The amount of singlet oxygen production is influenced by both the surface functional group and the duration of light exposure, with carboxylic acid, hydroxyl, aldehyde, and ketone groups playing crucial roles in this process.

Additionally, the rich surface chemistry of GQDs offers locations for loading therapeutic agents, making these materials promising candidates for targeted drug delivery. Due to their ultra‐small size (with lateral dimensions below 20 nm), GQDs possess the ability to traverse the blood–brain barrier [87]. This unique property makes GQDs specifically suitable for delivering drugs to the central nervous system. Further, for bioimaging and biosensing applications, the purity of GQDs is critical to attain the high sensitivity and detection efficiency [88, 89, 90]. Therefore, to ensure steady performance, several purification methods are employed, including size‐selective filtration, ultracentrifugation, dialysis coupled with salting‐out extraction, direct salting‐out, and acid/base treatments.

### 3.2 Optical Attributes of GQDs

GQDs, which are characterized by their lateral dimensions below 100 nm, exhibit quantum confinement effects in all three spatial dimensions. In contrast to other carbon‐based quantum dots (CQDs) that may demonstrate similar PL behavior, GQDs possess a separate crystalline structure [91]. Since the functional groups of the GQDs play a significant role in modifying the bandgap of these materials, the optical features of these materials can be effectively tailored via chemical modifications at their edges and on their surface. These edge and surface functionalities also facilitate electron–hole recombination near the border, which contributes to the observed PL emission. Additionally, introducing heteroatom dopants such as P, N, S, or F into the GQD structure can create defects that further enhance their PL QY (PLQY).

The optical characteristics of GQDs have been extensively studied using techniques such as PL spectroscopy and UV–visible absorption measurements [17, 92]. Based on these studies, it has been reported that GQDs display intrinsic PL characteristics that are absent in pristine graphene. Typically, the absorption spectra of GQDs reveal strong bands in the deep UV region and sometimes in the visible range. These spectral features can shift based on changes in the surface characteristics and morphology of the GQDs. The absorption bands at shorter wavelengths are generally associated with π–π* electronic transitions, while those at longer wavelengths correspond to *n*–π* transitions involving nonbonding electrons [93]. Several parameters, including particle size and shape, solvent environment, temperature, chemical composition, and surface functionalization, can influence the intensity and location of these absorption bands [94, 95]. The tunability of GQDs, along with the inherent low toxicity and biocompatibility of these materials, makes them a promising nanomaterial for various optical and electronic applications. Further, based on the synthesis approach that is used, surface modification, variation in size, and variation in the heteroatom dopants, the emission wavelengths and PL of GQDs can extend over a broad range [96]. A few reports have demonstrated GQDs emitting in the ultraviolet [97], blue [98, 99], green [100], yellow portions of the spectrum [101], and sometimes in the red portion of the spectrum as well. For instance, Tang et al. [92] reported emission over a wide range from 300 to 1000 nm from N‐GQDs. Further, variations in the synthesis approach can lead to different morphologies of GQDs, including edge structure, lateral size, and crystallinity, which directly influence the PL properties of these materials. Previous studies have indicated that GQDs exhibit superior photostability compared with other traditional QDs [20, 102]. The PL mechanism in GQDs is multifaceted, influenced by factors such as the degree of π‐conjugation, surface functionalization, particle size and shape, oxidation level, and the pH level of the medium [103, 104, 105, 106]. Although GQDs often exhibit low QYs, the QY values can be enhanced through appropriate heteroatom doping and surface engineering [107, 108, 109, 110, 111].

It has been observed that chemical modifications involving functional groups such as ‐COOH, ‐C‐O‐C‐, and ‐C = O can create surface oxidation states that strongly influence the PL emission characteristics of GQDs [108, 110]. For instance, Tetsuka et al. [60] demonstrated that amine‐functionalized GQDs that were synthesized through the oxidation of graphene sheets exhibited tunable optical properties. Likewise, Shen et al. [74] synthesized polyethylene glycol (PEG) diamine functionalized GQDs, yielding blue PL characteristics with a QY of 7.4%. The aromatic carbon edges of as‐synthesized GQDs also offer binding sites for further functionalization [74]. Luo et al. modified GQDs using aryl groups, leading to a PL shift from 418 to 447 nm and a sixfold increase in QY; these characteristics were attributed to enhanced π–π interactions between the GQD framework and aryl moieties [3, 112]. It was also noticed that the doping of heteroatoms into the GQD lattice leads to both structural and electronic modifications [113]. Codoping strategies enable synergistic effects by combining the properties of multiple dopants and enhancing the optical properties of GQDs [114, 115]. For example, Peng et al. [115] synthesized N‐, S‐codoped GQDs using citric acid and l‐cysteine; these materials demonstrated yellow‐green emission at 500 nm, in contrast to blue emission at 417 nm from undoped GQDs.

### 3.3 Biosafety and Toxicity Assessment of GQDs

The biosafety profile of GQDs is mainly determined by their physicochemical characteristics, including particle size, surface functionalization, surface charge, and synthesis method [116]. Due to the fact that GQDs are carbon‐based structures and exhibit a lower tendency to release toxic ions, these materials are considered more biocompatible than other nanomaterials and metallic QDs (e.g., CdSe or PbS). Further, GQDs also exhibit intrinsic fluorescence, the aqueous stability of GO, along with broad chemical adaptability. Although GO and other carbon‐based nanomaterials have demonstrated significant potential for use in biomedical applications, including diagnostics [117, 118, 119, 120, 121, 122, 123, 124], biosensing [125, 126, 127, 128, 129, 130, 131, 132], and drug delivery [133, 134, 135, 136], recent research efforts have focused on assessing the biosafety of GQDs [137, 138, 139, 140, 141, 142, 143, 144, 145]. It should be noted that conflicting findings have emerged regarding the cytotoxicity of GQDs. For instance, several studies report the excellent biocompatibility and low toxicity of GQDs [146, 147, 148, 149], while other studies indicate potential cytotoxicity of GQDs, mainly at higher concentrations or with specific surface modifications. Efforts to better understand the interactions of GQDs with biomolecules such as DNA, proteins, and cell membranes are continuing to be pursued. Several reports mention that GQDs exhibit lower cellular toxicity due to their superior water solubility [150, 151, 152]; these findings stand in contrast to those obtained from metal‐based nanoparticles and other carbon‐based nanoparticles [152, 153, 154]. Unlike metallic nanoparticles, the use of GQDs is not associated with the formation of toxic metal deposits. Several reports suggest that smaller GQDs do not disrupt lipid membranes; this characteristic may facilitate the use of these materials for biological applications [153]. A few in vivo studies also showed rapid renal clearance of GQDs without significant hepatic accumulation; this characteristic indicates that these materials may have an appropriate biosafety profile [155]. It has also been observed that surface functionalization can modify the properties of GQDs; for instance, amide‐, amine‐, and carboxyl‐modified GQDs demonstrate low toxicity even at high concentrations [156]. In contrast, hydroxyl‐functionalized GQDs may encourage ROS generation at elevated doses; however, recent findings suggest that ROS production may depend on pre‐existing cellular dysfunction [157].

Recently, a report by Tabish et al. [158] indicated that small‐diameter GQDs at microgram‐to‐milligram concentrations show minimal toxicity in rodent cell lines. However, contradictory studies report adverse effects of these materials, highlighting the need for further investigation [158, 159]. For example, Wang et al. [160] observed cytotoxicity in red blood cells exposed to N‐GQDs; it should be noted that their effects were comparable to those of GO. Lee et al. [161]identified enzymatic degradation (e.g., by HRP) and renal excretion as key detoxification pathways associated with GQDs in mice. Further, PEG conjugation (PEGylation) has been proposed as an effective strategy to reduce the toxicity of GQDs [161]. Previous studies have shown that PEGylated GQDs exhibit reduced cytotoxicity; no significant cell death was observed in HeLa cells at concentrations up to 160 µg/mL. The favorable biocompatibility of GQDs is further attributed to the small size, high oxygen content, and efficient renal clearance of these materials [162]. Furthermore, heteroatom‐doped GQDs (e.g., GQDs doped with N, S, or P) show enhanced biocompatibility and minimal cytotoxicity [12, 16, 163, 164, 165, 166, 167, 168]. Recent preclinical and clinical studies indicate the negligible toxicity of GQDs at cellular and tissue levels [169]. Assessments of cell viability, apoptosis, lactate dehydrogenase release, and ROS generation further support the safety profile of GQDs. Unlike GO, GQDs do not induce significant toxicity in mice; any observed adverse effects likely result from aggregation rather than from the GQDs themselves [170, 171]. Repeated‐dose toxicity studies further confirm the low in vivo toxicity of GQDs [172].

While these findings provide valuable insights, there remains a need for more comprehensive research to fully understand the biocompatibility and toxicological effects of GQDs. The biosafety profile of GQDs can be influenced by multiple factors, including size, surface chemistry, concentration, and redox state [173]. For instance, the concentration and redox state of GQDs influence cell viability, while the size of GQDs determines the ability of these materials to penetrate biological barriers. Although GQDs hold significant promise for a wide range of applications, a detailed biosafety assessment is essential to ensure the safe use of GQDs in biomedical settings. Notably, surface‐enhanced infrared absorption spectroscopy has been utilized in toxicological studies to assess the interactions of GQDs with their environment [174, 175, 176, 177]. GQDs demonstrate considerable potential for biomedical applications due to their favorable biocompatibility and low cytotoxicity in vitro and in vivo. However, further research into GQDs is needed to optimize functionalization strategies, fully elucidate their safety profile, and validate their use in clinical settings.

### 3.4 Nanotoxicology Assessment of GQDs

In recent years, GQDs have attracted significant scientific interest due to their exceptional potential across a wide range of applications. However, before GQDs can be widely utilized, particularly in biomedical applications such as biosensing, bioimaging, and targeted drug delivery, the nanotoxicological profile of these materials must be thoroughly understood. While GQDs offer considerable promise, their biosafety profile must be validated to enable their use in practical applications. An increasing number of studies have evaluated the toxicity of GQDs, both through theoretical and experimental studies [138, 139, 178, 179, 180]. The toxicity of materials toward living organisms is typically evaluated through controlled laboratory studies using both in vitro (cell‐based) and in vivo (whole‐organism) experimental models. In vitro toxicity assessments, commonly referred to as cytotoxicity tests, are typically conducted using cultured cells, whereas in vivo studies involve entire organisms [138, 141, 170]. Recently, various types of heteroatom‐doped GQDs, including N‐ and B‐doped GQDs (N‐GQDs and BGQDs), have been evaluated using cell lines (e.g., HeLa, A549, MCF‐7) and using models such as mice and zebrafish [139, 142]. These studies reveal that GQDs exhibit relatively low toxicity and excellent biocompatibility as compared with other nanomaterials (e.g., GO and CNTs) and conventional semiconductor QDs. These characteristics make GQDs a promising candidate for biomedical applications. Nevertheless, the toxicity and biocompatibility of GQDs can vary depending on the type of GQDs, concentration of GQDs, synthesis method, surface chemistry, dopant, and generation of ROS. For instance, Zhang et al. [181] reported that the GQDs are biocompatible up to a concentration of 0.5 mg/L; some studies reported a safe threshold around 200 µg/L. Hai et al. [182] observed a greater level of cell viability and good biocompatibility in GQDs prepared via bottom‐up methods as compared with GQDs prepared via top‐down approaches. In another study, Ouyang and coworkers mentioned that GQDs <10 nm in size were found to be less toxic compared with GQDs of larger size and nano graphene sheets [183]. Further, Wang et al. [184] reported that elemental doping and the presence of functional groups on GQD surfaces can significantly improve the compatibility of GQDs with biological systems. Hu et al. [185] reported that while GQDs are considered relatively safe, the risk of ROS‐induced toxicity cannot be ruled out. To address this consideration, surface modification strategies such as PEGylation have been reported to be effective in reducing the oxidative stress and enhancing the biocompatibility of GQDs [185, 186]. Despite the progress in assessing the toxicology of GQDs, a comprehensive understanding of the toxicological behavior of GQDs remains incomplete; there is an urgent need to develop standardized protocols for toxicity testing involving GQDs before these materials are used in the clinical setting.

## 4 Bioimaging and Drug Delivery Applications of GQDs

GQDs have emerged as highly promising nanomaterials in the fields of bioimaging and drug delivery due to their exceptional optical properties, biocompatibility, large surface area, physiological stability, and ease of functionalization [87, 187, 188, 189, 190, 191]. Due to their strong PL, tunable PL, high photostability, and minimal cytotoxicity, GQDs are ideal candidates for fluorescent bioimaging; because of these characteristics, GQDs can serve as an alternative to traditional organic dyes and toxic metal‐based QDs. GQDs can emit light across a broad spectrum and are capable of multicolor imaging, enabling real‐time tracking of cellular processes with high spatial and temporal resolution. Additionally, their small size facilitates deep tissue penetration, while their ability to be excited under near‐infrared (NIR) light minimizes background autofluorescence and photodamage to cells. These features enhance the sensitivity and specificity of imaging, particularly for cancer detection and neurological studies. Moreover, the functional groups on the GQD surface may be modified via straightforward conjugation approaches with targeting ligands, antibodies, or peptides in order to enhance the selectivity of GQDs toward specific tissues or cellular receptors [192]. As a result, GQDs serve not only as imaging agents but also as multifunctional platforms for simultaneous diagnosis and therapy, paving the way for use in nanotheranostic applications. In addition to bioimaging and drug delivery, several studies on GQDs have already demonstrated the functionality of these materials in various optoelectronics and biomedical applications, including antibacterial, biosensing, PDT, photodetector, light emitting diode, rechargeable battery, photovoltaic device, supercapacitor, thermoelectric device, and photocatalysis device applications [193, 194, 195, 196, 197, 198]. The following subsections describe the use of GQDs in the development of bioimaging and drug delivery systems.

### 4.1 Bioimaging Applications

Early detection of disease is crucial for improving patient outcomes and reducing patient morbidity. This consideration motivates researchers to create highly sensitive QDs with exceptional specificity and minimal toxicity. GQDs stand out by exhibiting exceptional optical and electronic properties that are useful for biomedicine and photonics applications. The GQD is a zero‐dimensional (0D) nanomaterial that has attracted attention for use in several types of biomedical applications. In addition to their ultra‐small dimensions, GQDs are nontoxic, biocompatible, fluorescent in a tunable manner, and water soluble. Due to their ability to withstand exposure to light and retain stability, GQDs exhibit useful performance in several types of biomedical applications. The fluorescence of GQDs, which can be modified via doping (summarized in Table 2), adds another layer of versatility, allowing for precise detection and imaging in biological systems.

**TABLE 2:** Comparative analysis of the fluorescence properties of GQDs for bioimaging applications.

Nanomaterials	Size (nm)	Quantum yield	PL color	Emission (nm)	Excitation (nm)	References
GQDs	2–5 nm	55%	Green	535	420	[199]
GQDs–oleylamine (OA)	15–35 nm	0.64	Green	490	405	[200]
GQD–CoOOH nanoflakes	2–7 nm	–	Red	660	480	[201]
N–B‐GQDs	∼5 nm	1.0%	Bright fluorescence	1000	368	[184]
Gd_2_O_3_/GQD nanocomposites	3.93 ± 0.53 nm	–	Green	515 nm	365 nm	[202]
Amino‐N‐GQDs	7.1 ± 0.6 nm	0.55	‐	678	630	[203]
N‐GQDs	8.0 ± 0.6 nm	0.38	Red	800	365	[204]
GQD–PEG	15	18.8	Green	518	400	[205]
N‐GQD/N–S‐GQD	5.5–3.9 avg.	60	Cyan, green	800–890	425/448	[68]

Abbreviations: CoOOH: cobalt oxyhydroxide, Gd_2_O_3_: gadolinium oxide, PEG: polyethylene glycol.

**FIGURE 4:**
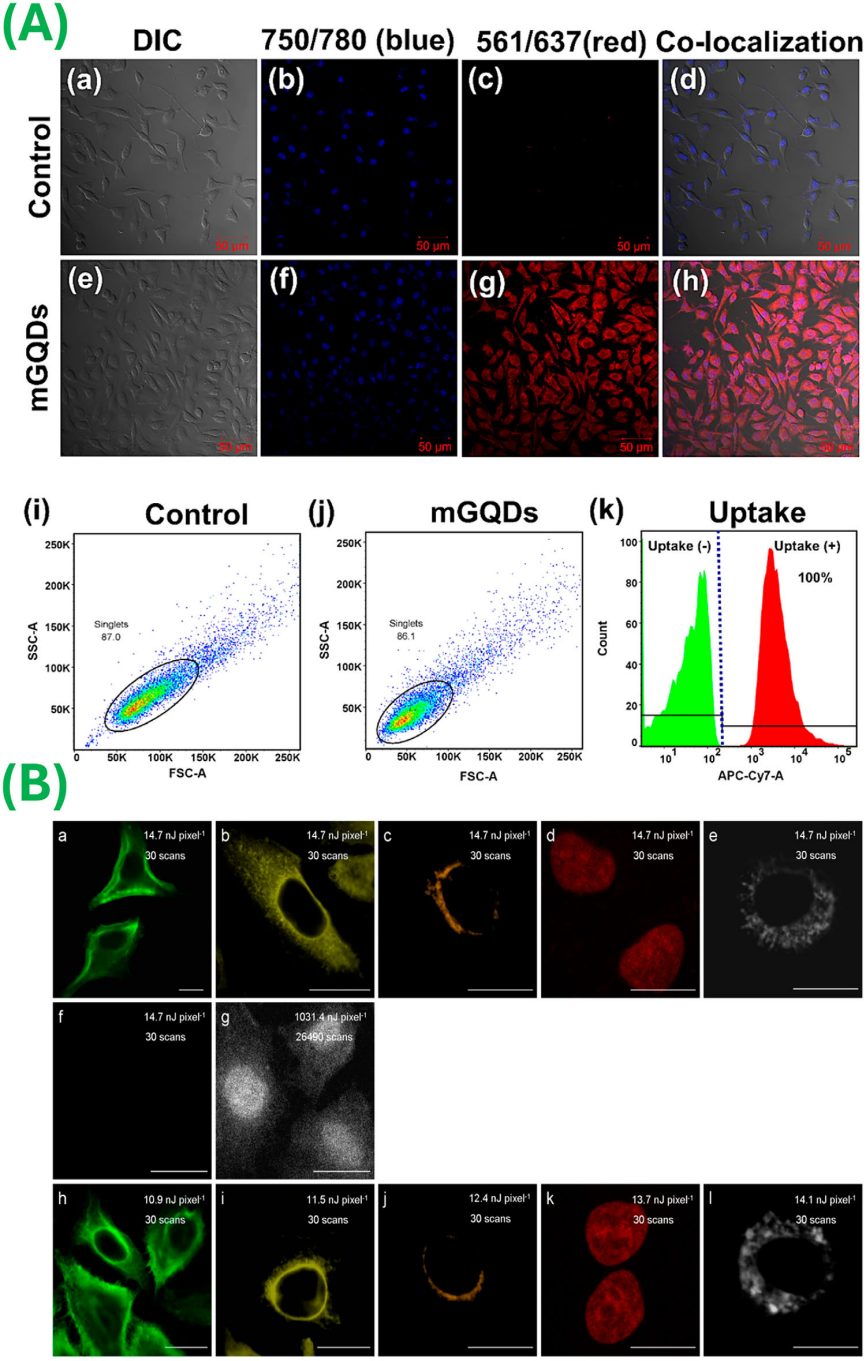
Bioimaging application of GQDs. (A) Bioimaging and cellular uptake studies of L929 cells treated with mGQDs after 24 h (reproduced with permission from Ref. [67], Copyright 2017 American Chemical Society). (B) Two‐photon luminescence images of N‐GQD–PEI–Ab‐treated A549 cells captured at different excitation powers. (Reproduced with permission from Ref. [204], Copyright 2020 American Chemical Society.)

Due to their photostability, GQDs are highly stable when exposed to light, which enables the use of these materials for several biomedical situations. Moreover, the water solubility of GQDs makes it straightforward to incorporate these materials into aqueous environments, enabling straightforward interactions with biological components. Pan et al. [17] made the first demonstration of the fluorescent characteristics of GQDs in 2010; since that time, several efforts have been made to develop fluorescent probes for in vitro imaging, in vivo tumor imaging, and evaluating cellular dynamics. One of the authors has demonstrated a hydrothermal method to create ultrafine GQDs emitting strong blue light and proposed a mechanism involving the breakdown of epoxy chains into GQDs. These materials could be useful for applications such as electronic devices and biological labeling. Two‐photon (TP) fluorescent probes based on these materials may prove useful for biological research and biomedical diagnostics. The probes must exhibit exceptional durability, withstand photodegradation, demonstrate superior imaging depth, and overcome constraints imposed by autofluorescence. The scarcity of probes with these characteristics to this point has hindered the ability to perform real‐time cell imaging in thick tissues, restricting imaging to a nominal depth threshold of ∼1000 µm [199, 206]. Evaluation of the photophysical characteristics of GQDs has revealed their nonblinking behavior and notable TP cross‐section. Consequently, GQDs can be efficiently stimulated via NIR irradiation, which obviates the need for deleterious high‐energy light sources. This characteristic not only aids in suppressing interference from endogenous background fluorescence but also facilitates enhanced penetration into biological matrices [207]. These characteristics, coupled with the low scattering rates of GQDs, contribute to the high signal‐to‐noise imaging ratio values associated with these materials; as such, GQDs are a viable candidate for intracellular and in vivo imaging applications [200, 208]. Feng et al. [201] discussed the characterization of the TP properties of GQDs; they demonstrated NIR emission at 660 nm under excitation by 810 nm femtosecond pulses and a cross‐section value of 25.12 GM. They introduced NIR GQDs with TP fluorescence properties, enabling the construction of a nanoprobe for bioimaging and direct detection of endogenous ascorbic acid (AA) in living cells. The nanoprobe exhibited high sensitivity (LOD of 270 nM), selectivity, and photostability, enabling deep‐tissue imaging. Singh et al. [199] explored the synthesis of TP excitable GQDs derived from neem plant root extracts using a straightforward solvothermal method. GO sheets were initially generated, which underwent further breakdown to yield well‐dispersed GQDs. The resulting GQD dispersion exhibited long‐term stability and homogeneity at room temperature for extended periods. Naumov et al. [209] focused on developing NIR emissive GQDs with high biocompatibility to address concerns regarding the clinical use of carbon nanomaterials. Various synthetic methods, including bottom‐up approaches and top‐down approaches with doping strategies, produced GQDs with sizes ranging from 2 to 5 nm and desirable spectral properties. N, S, or rare‐earth metal doping enhances NIR emission, while oxygen‐containing functional groups facilitate water solubility and cellular uptake. The GQDs demonstrated a high level of biocompatibility up to a concentration of 1–2 mg/mL and degraded in cell culture within 36 h. The materials exhibited visible and NIR fluorescence, with QYs up to 60%. In vivo studies in live mouse models confirmed the NIR imaging capabilities of the materials; fluorescence was demonstrated in the liver, spleen, and kidneys. The findings suggest that biocompatible NIR‐emissive GQDs could serve as imaging and drug delivery agents in both in vitro and in vivo applications. Conventional NIR fluorescence imaging, commonly referred to as NIR‐I, operates within the spectral range of 750–900 nm [210]. Srivastava et al. [67] synthesized materials via a microwave‐assisted green route from Mangifera indica leaf extracts, which exhibited red luminescence in the NIR range (650–750 nm) and quantum‐sized dimensions (2–8 nm); they referred to these materials as mGQDs. The materials demonstrated high cellular uptake and biocompatibility with L929 cells even at a concentration of 0.1 mg/mL (Figure 4A). Additionally, mGQDs can serve as NIR‐responsive fluorescent bioimaging probes and temperature‐sensing agents (10–80°C); these materials exhibited stable signals after multiple cycles. The fluorescence intensity value of the material decreased with increasing intracellular temperature (25–45°C), indicating the potential use of the material for biomedical nanothermometry applications. Wang et al. [184] developed N and B dual‐doped N–B‐GQDs with an ultra‐small size of ∼5 nm as contrast agents for NIR‐II imaging (1000–1700 nm); they evaluated the in vivo imaging efficacy of the materials in mice. N–B‐GQDs exhibited functionality for use in photothermal therapy, including the elimination of cancer cells in vitro and suppression of tumor growth within a murine glioma model. The materials demonstrated a prolonged blood half‐life, a safe profile, and rapid excretion, making them suitable for in vivo biomedical use.

Magnetic resonance imaging (MRI) is a commonly used bioimaging technique due to its remarkable sensitivity, penetration depth, resolution, and capacity for precise tissue imaging. The operational principles of MRI rely on the excitation and subsequent relaxation processes of hydrogen nuclei, which are plentiful in the aqueous and lipid components of biological tissues [211]. MRI contrast agents, which are used for enhancing image contrast, are typically categorized into two main classes, T1 and T2 agents, based on their effects on the longitudinal (R1) and transverse (R2) relaxation rates, respectively. These agents play an important role in improving the specificity and accuracy of MRI diagnostics, enabling the precise localization and characterization of disease‐containing tissues [202, 203, 212]. T1 contrast agents are categorized as positive contrast agents and have found extensive usage in clinical applications as blood agents, extracellular agents, and hepatobiliary agents. Due to their ability to produce brighter MR imaging, positive T1 contrast agents offer a benefit with regard to spatial resolution [213]. Gadolinium oxide (Gd_2_O_3_) nanoparticles, which are T1 contrast agent materials, are recognized for their favorable positive contrast efficiency and low toxicity [214, 215]. Surface modification is crucial for Gd_2_O_3_ nanoparticle dispersion and Gd ion leakage prevention. Conventional surface modification methods involve the use of organic surfactants or silica coatings. Enhancing the water permeability of coatings is essential for T1 contrast agents. It should be noted that a higher silica shell porosity improves R1 relaxivity. Phase transfer agents with additional functionalities are preferred for multimode imaging. GQDs demonstrate robust colloidal and PL stability within neutral to alkaline solutions (pH 5–12) [216]. The functional groups on GQD edges may be modified to improve MRI performance and enhance water penetrability. The GQD coating can also impart fluorescence, enabling the formation of dual‐functional nanocomposites for MRI and cell labeling.

Wang et al. [202] developed a dual‐functional imaging contrast agent via coating of Gd_2_O_3_ nanoparticles with TP GQDs. Unlike other types of dual‐modal contrast agents, this approach involved a simplified surface modification approach. The GQD coating enhanced the water penetrability characteristics of the material, which enhanced the MRI performance of the Gd_2_O_3_/GQD nanocomposites. The presence of the edge functional groups on the GQDs improved the colloidal stability and biocompatibility of the materials. Additionally, the Gd_2_O_3_/GQD material exhibited TP excitation properties, reduced photo‐bleaching, and deep tissue penetration functionality for bio‐imaging. Kuo et al. [204] fabricated N‐GQDs via polypropylene membranes to ensure a uniform distribution of oxygen (O) and N, resulting in materials that exhibited high crystallinity and possessed a size of 0.9–8.4 nm. The synthesized N‐GQDs demonstrated exceptional TP properties across the ultraviolet (UV) to NIR excitation spectra without spectral overlap (Figure 4B). These advantageous properties are attributed to the enhanced charge‐transfer functionality that was facilitated by the π‐conjugated system, and heightened electron donation within the N‐atom‐induced polymers that were conjugated with N‐GQDs.

With the development of improved T2‐weighted MRI imaging, magnetic nanoparticles (MNPs) are finding greater use as imaging agents. By fine‐tuning the size, surface properties, stability, and magnetism of the materials, researchers aim to develop novel nanoparticles for medical imaging applications. Branca et al. [217] explored the development of MNPs for T2‐weighted MRI by optimizing parameters such as size, surface properties, and magnetization. Iron‐based nanoparticles with various surface coatings were evaluated in terms of their efficacy as contrast agents. They observed that the silica‐coated iron nanocubes demonstrated the highest relaxivity values (628 s^−1^ mM^−1^). Parker et al. [218] described a comprehensive theoretical framework to determine the minimum concentration of T2 contrast agents necessary for optimal image contrast. The analytical model was versatile and applicable across various T2‐type agents and delivery methods, requiring only a few easily obtainable parameters. Applying the model, the contrast generated by superparamagnetic ferumoxide and ferritin, an iron storage protein, was predicted and experimentally validated via Feridex and ferritin suspensions in phantoms. The model was utilized to compare the contrast efficacy of metal ions in two clinically approved T1‐ and T2‐type contrast agents. Xiao et al. [99] provided an overview of MRI contrast agents, categorizing them based on composition, administration route, magnetic properties, and imaging applications. Paramagnetic agents enhance signal intensity on T1‐weighted images, while negative agents decrease signal intensity values on T2‐weighted images. Chelating agents aid in excretion and reduce toxicity. The agents are administered orally or intravenously; they are categorized into blood, extracellular fluid, and target/organ‐specific types based on biodistribution and intended application. Contrast agents have been developed for imaging liver disease, organ targeting, inflammation, and tumor detection.

### 4.2 Drug Delivery Applications

GQDs, due to their unique structural and optical properties, have emerged as a promising platform for drug delivery applications and have shown excellent performance for theranostic applications [219, 220]. Due to their unique physio‐chemical characteristics, such as large surface area, compatibility with straightforward surface modification methods, and enhanced cellular uptake, these nano‐sized carbon‐based materials have been considered for applications in drug delivery, photothermal, photodynamic, and nano‐theranostic applications [82, 221]. Further, GQDs can act as efficient nanocarriers for a wide variety of therapeutic agents, nucleic acids, proteins, and small compounds [205, 222, 223]. The large surface area of GQDs makes it possible to load a large amount of therapeutic molecules onto the surface of the materials; GQDs may be used to deliver these moieties in a target‐specific manner. The ease of surface modification enables the incorporation of various biomolecules, polymers, and nanoparticles to improve the biocompatibility, solubility, and targeting capabilities of the materials [221]. Recent studies have demonstrated the versatility of GQDs for drug delivery (summarized in Table 3). GQDs have been used to deliver several types of materials, such as siRNA, miRNA, targeted genes, and multiple drug delivery agents, in a target‐specific manner [219]. For instance, GQDs have been functionalized with targeting ligands to enhance the selective accumulation of the materials in tumor tissues, thus improving the therapeutic efficacy of the encapsulated anticancer drugs. Recently, Wang et al. [224] described the application of miRNA‐conjugated GQDs for suppressing breast cancer development genes. Campbell et al. [225] explored the multifunctional characteristics of GQDs; they demonstrated the fabrication of biocompatible glucosamine‐based GQDs as a multifunctional imaging and delivery platform via the conjugation of hyaluronic acid (HA), for targeting CD44 receptors, and ferrocene (Fc) drug. The Fc–GQD–HA material showed an enhanced ROS‐mediated cytotoxic response in HeLa cells, while exhibiting an absence of toxicity at 1 mg/mL to noncancerous HEK‐293 cells.

**TABLE 3:** Summary of the drug delivery application of different GQDs.

Nanomaterials	Size (nm)	Drugs loaded	Target cells	Cell viability (%)	References
Fc–GQD–HA	3–5 nm	Ferrocene (Fc)	HeLa cells, HEK 293	80	[225]
GQD–PEG	15	Doxorubicin (Dox)	–	–	[205]
N‐GQDs and Nd–N‐GQDs	4.0 ± 0.6 and 3.8 ± 0.9 nm	siRNA	HeLa	∼70–80	[226]
GQD–RGD	4	Dox	DU‐145, PC‐3, and MC3T3‐E1	45–60	[227]
CS–GQDs nanocomposite	3.82 ± 0.63 nm	Latanoprost	Human corneal epithelial (HCE) cells	>80%	[228]
N‐GQDs–APTES	4	Dox	Nucleus	17	[229]
Fe_3_O_4_@PEG@GQD	129 ± 10	Dox	MCF‐7	∼90–95	[230]
GQD‐RGD	3.7	Dox	U251 glioma cells	70.9	[231]
GQD–Cyc–HCl	5	Berberine hydrochloride (BHC)	L929 cells, HeLa cells, and MDA‐MB‐231 cells	∼50	[232]
N‐GQD	7–17	Methotrexate	MCF‐7	∼80	[65]

Abbreviations: Fc: ferrocene, Nd: neodymium, RGD: arginyl‐glycyl‐aspartic acid, APTES: (3‐aminopropyl)triethoxysilane, Fe_3_O_4_: iron oxide, Cyc‐HCl: cysteamine hydrochloride.

**FIGURE 5:**
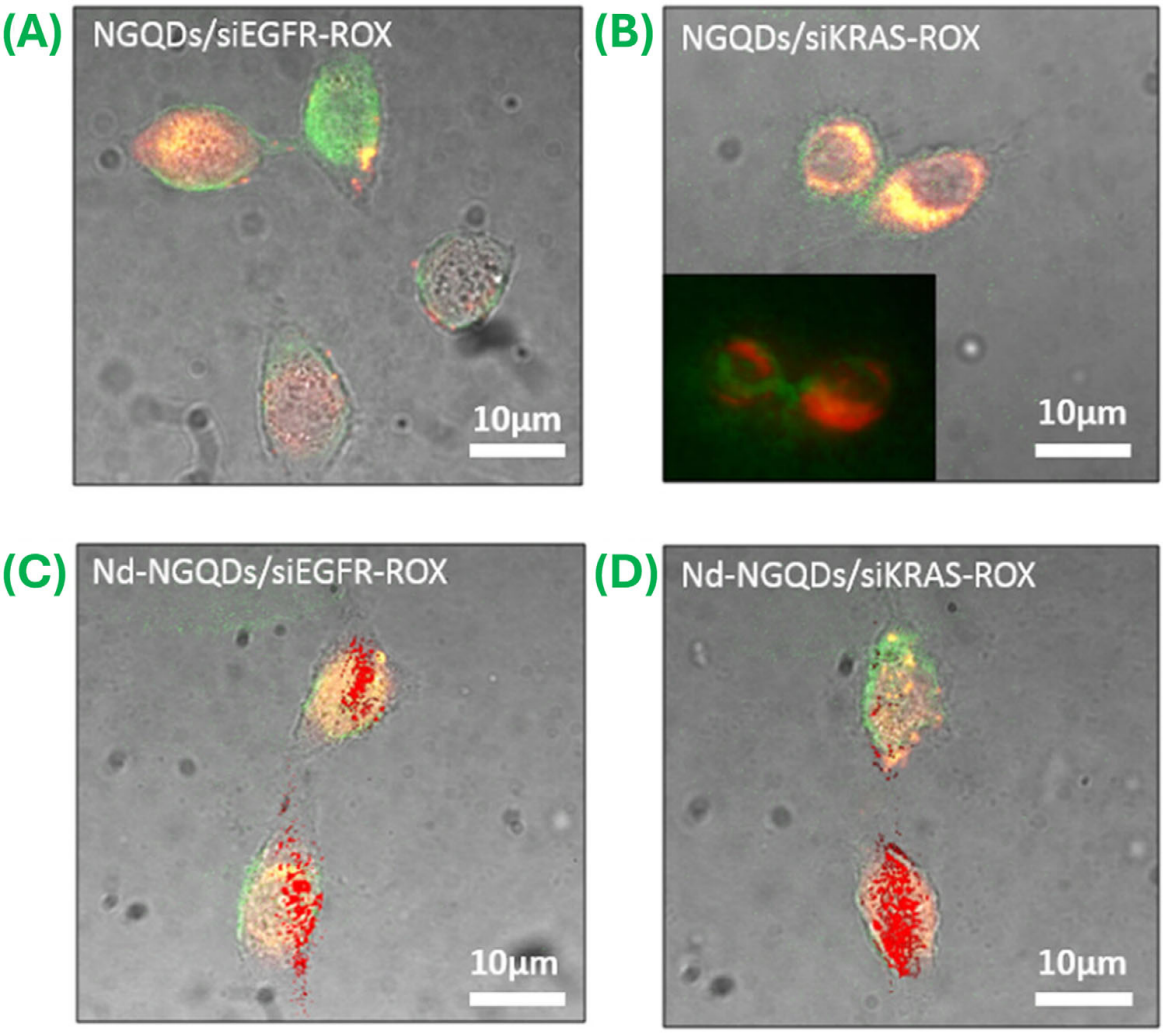
Drug delivery application of GQDs. Illustration of bright‐field/visible fluorescence confocal overlay images of HeLa cells treated with (A) N‐GQDs/siEGFR–ROX, (B) N‐GQDs/siKRAS–ROX, (C) N‐GQDs/siEGFR–ROX, and (D) Nd–N‐GQDs/siKRAS–ROX for 12 h. (Reproduced with permission from Ref. [226], Copyright 2023 American Chemical Society.)

Valimukhametova et al. [226] considered the utilization of biocompatible N‐ and neodymium‐doped graphene QDs, referred to as N‐GQDs and Nd–N‐GQDs, respectively, as a multimodal delivery platform. They investigated the efficacy of N‐GQDs and Nd–N‐GQDs that were conjugated with Kirsten rat sarcoma virus, as well as epidermal growth factor receptor siRNA, against various types of cancer. Interestingly, they demonstrated efficient cellular uptake of the GQD/siRNA complex and successful siRNA transfection into HeLa cells (Figures 5A–D). In addition, effective protein knockdown took place within HeLa cells at nanomolar concentrations of siEGFR and siKRAS, which confirmed the therapeutic potential of the GQD/siRNA complex. A key advantage of employing GQDs is their ability to facilitate stimulus‐responsive drug release. The surface of the materials can be engineered with stimuli‐responsive moieties, enabling the controlled release of drugs in response to various environmental cues (e.g., pH or enzymatic activity) [228, 230]. This targeted delivery approach allows therapeutic agents to reach desired sites, improving their efficacy while potentially reducing unwanted side effects. Sheng et al. [233] described the synthesis of a novel CS/GQDs/cytarabine (Cyt) platform, Cyt‐loaded GQDs wrapped with chitosan (Cs), for pH‐responsive burst drug release. Javadian et al. [230] described the fabrication of superparamagnetic Fe_3_O_4_@PEG@GQD nanocarriers for the targeted delivery of doxorubicin (Dox) to cancer cells; the authors noted reduced toxicity (to normal cells) and a high drug loading content level (27%) along with superior superparamagnetic properties. The material demonstrated a pH‐dependent drug release profile, with a higher release rate at pH 5.0 than at pH 7.4. Several research groups demonstrated significant enhancement in the therapeutic efficacy of chemotherapeutic drugs through selective enzyme‐responsive drug release into the tumor microenvironment [228, 234]. For instance, Kumara et al. [228] reported the lysozyme enzyme‐responsive delivery of an antiglaucoma drug, latanoprost, through a Cs‐decorated GQD‐based nanocarrier via a reverse‐switching PL mechanism. Wibrianto et al. [235] synthesized highly fluorescent N, copper, and S‐doped CQDs (N, Cu, S‐CQDs), and reported an excellent QY of up to 84%. Further, they conjugated Cu, N, S‐CQDs using glucose oxidase (GOx) and camptothecin (CPT) to generate a multifunctional Cu, N, S‐CQDs@GOx/CPT nanocarriers that exhibited an elevated therapeutic response via enhanced production of hydroxyl (•OH) radicals and H_2_S gas. Via an enzymatic cascade reaction, including H_2_O_2_ self‐formation, they demonstrated the ROS generation features of the nanocarrier. They also proposed potential applications of the nanocarrier in gas therapy‐based and chemodynamic therapy‐based treatment of cancer.

The ability of GQDs to serve as both imaging agents and drug delivery vehicles makes them particularly well suited for theranostic applications [236]. The strong PL and tunable emission wavelength of GQDs allow them to be used as fluorescent probes for bioimaging; their large surface area and ease of surface modification enable the loading and targeted delivery of therapeutic agents [237]. This dual functionality of GQDs as both drug delivery vehicles and imaging agents can facilitate the development of theranostic platforms, in which diagnosis and treatment are integrated into a single platform. GQDs can be structurally engineered to carry both anticancer drugs and target‐specific tumor markers; the materials carried by the GQDs may enable the visualization of the tumor site and the controlled release of the therapeutic payload in a target‐specific manner [238]. The optoelectronic properties of GQDs can be modified by changing their size and surface functionality. In general, smaller GQDs exhibit higher optical absorption and PL due to their wider band gap values. On the other hand, by increasing the size of GQDs, the band gap can be decreased and the PL can be shifted to a longer wavelength [239]. In order to exhibit PL at the desired wavelength (usually a wavelength in the NIR region), one can modulate the size of the GQDs and band gap accordingly. Recently, Gomez et al. [240] described synthesizing organotin‐decorated N‐GQDs for use as dual‐responsive agents, which can be used for molecular imaging as well as the treatment of triple‐negative breast cancer. The authors demonstrated high cellular uptake, enhanced fluorescence of N‐GQDs–FBA–Sn, and a high cytotoxic response against MDA–MB‐231 breast cancer cell lines. Haider et al. [241] demonstrated the application of peptide‐conjugated GQDs against placenta‐specific protein 1 (PLAC‐1) overexpressed colorectal cancer cells (CRC). The peptide‐conjugated GQDs demonstrated the capability for selectively targeting the overexpressed antigen PLAC‐1 on HT‐29 and HCT‐116 cells, with an enhanced cytotoxic response. Recently, Hojjati et al. [242] described the synthesis of a novel drug delivery nanoplatform based on magnetic core–mesoporous silica nanoparticles (MMSN) capped using GQDs and conjugated regorafenib drug, and demonstrated the cytotoxic potential of this material against the SW‐480 CRC line. The results revealed the excellent theranostic capabilities of MMSN–REGO/GQDs as well as enhanced superparamagnetic properties, indicating the potential use of this material as an MRI contrast agent with improved therapeutic efficacy.

GQDs, due to the presence of a *sp* ^2^ hybrid carbon framework as well as the occurrence of carboxyl groups along the edges, exhibit excellent single oxygen generation capabilities as well as efficacy for photodynamic and photothermal therapy [243]. Moreover, the presence of an electron‐conducting *sp* ^2^ hybrid network, enhanced photostability, impressive water dispersibility, and wide pH stability allow these materials to be used for various PDT applications. Recently, Yang et al. [244] described the synthesis of acidity‐activated GQDs‐based nanotransformers (GQD‐NT) for tumor imaging over a long duration and repeated PDT through stimuli‐responsive activation of the photosensitization functionality. The Arg–Gly–Asp peptide‐conjugated GQD‐NT selectively targets tumor tissues with prolonged tumor retention, generates a mild hyperthermia response on irradiation, and exhibits an effective PDT response. Kang et al. [245] demonstrated the therapeutic potential of hybrid GQD‐loaded macrophages against A549 human lung cancer cells. The authors described the enhancement of ROS by GQD‐loaded macrophages in a 3D culture model and a tumor‐homing effect in a xenograft mouse model.

In another study, riboflavin (Rf)‐conjugated amine‐functionalized N‐GQDs (am‐N‐GQD–Rf) were synthesized and investigated for inducing the enhanced PDT response by TP PDT‐based intraparticle fluorescence resonance energy transfer in various types of cancer cell lines [246]. The am‐N‐GQD‐Rf exhibited superior single oxygen generation on irradiation by UV and NIR light, resulting in a reduction in cell viability in various types of cancer cells via one‐photon and TP‐based PDT responses. Several studies highlight the potential of GQDs in drug delivery applications; ongoing research is focused on optimizing the design, surface functionalization, and biocompatibility of the materials to enhance their therapeutic efficacy and safety profile. The translation of GQD‐based drug delivery systems from the laboratory to clinical settings remains a promising area of investigation. Recently, Wu et al. [247] developed peptide‐loaded GQDs‐decorated luminescent porous Si (GQDs@PSi) for the fabrication of a stimuli‐responsive smart dressing for theranostic management of diabetic wounds. It was observed that the functionalization of GQDs on the PSi surface considerably enhanced the loading capability and offered the capability for loading insulin and epidermal growth factor to facilitate the diabetic wound healing process. In vitro and in vivo results confirmed that the developed smart dressing healed diabetic wounds more quickly, with enhanced proliferation and migration of cells [247].

Shen et al. [248] used MD simulation and free energy calculation studies to examine the structural and thermodynamic properties of deoxyadenosine and Dox drug translocation into the cell membrane with the assistance of GQDs. Using the simulation approach, they noted that GQDs can aid the movement of these drugs into the lipid membrane within nanoseconds and without any significant distortion in the cell membrane structure. They indicated that GQDs of optimal size could facilitate the drug delivery process by dropping the translocation free energy required for access via the cell membrane. More recently, the same group demonstrated that the drug delivery route for Dox could be split into two phases; GQDs and Dox initially aggregated into a cluster, and then the aggregates entered the cell membrane. GQDs offer a versatile and efficient platform for drug delivery due to their high surface area, excellent biocompatibility, and tunable surface functionalities. Their ability to transport and release therapeutic agents in a controlled and targeted manner offers great promise for enhancing treatment efficacy while minimizing the side effects.

## 5 Conclusions and Future Remarks

This review considers the recent advances in GQD research, particularly on the use of GQDs for bioimaging and drug delivery applications. In the first section, we discussed various bottom‐up and top‐down synthesis processes to prepare heteroatom‐doped and undoped GQDs, as well as the advantages and disadvantages of these approaches. They exhibit notable PL characteristics that make them valuable for use in bioimaging applications. GQDs have attracted significant attention in the field of bioimaging due to their ability to emit light across a wide range of wavelengths, offering real‐time visualization of biological processes. Their high surface area and ability to be functionalized with various chemical groups further enhance their utility in biological settings, allowing for imaging of cellular structures and tissues. In contrast to other nanoparticles, GQDs exhibit the ability to efficiently penetrate biological membranes with good biocompatibility.

Despite the attractive properties of GQDs, several challenges must be tackled to fully utilize these materials for bioimaging and drug delivery applications. One of the key barriers is the development of cost‐effective, scalable, and sustainable synthesis approaches that permit large‐scale production of GQDs without compromising their performance. Conventional methods, such as arc discharge and chemical vapor deposition, are associated with high costs and limited scalability. Recent efforts have considered more sustainable procedures, such as the hydrothermal approach, which employs high temperature and pressure in the presence of water to create GQDs. This method is cost effective, yields high‐quality GQDs, and is environmentally friendly, making it an ideal candidate for industrial‐scale production. Additionally, modifications to the graphite precursor using strong acids have been shown to substantially improve the yield of GQDs. These greener synthesis methods represent a key step to overcome the scalability limitations of conventional methods and increase the commercial feasibility of GQDs for bioimaging and drug delivery applications.

In addition to synthesis challenges, another significant barrier lies in optimizing the fluorescence and QY characteristics of GQDs, which are influenced by numerous factors, including size, surface functionalization, and the presence of functional groups or defects on the GQD surfaces. These factors can trigger extensive variation in the intensity, stability, and wavelength of the emitted light, complicating the use of the materials for bioimaging applications. For example, it is necessary to better understand how surface alterations impact the PL properties of GQDs. Surface modification with oxygen‐containing functional groups and doping GQDs with other materials play a crucial role in altering the emission and QY characteristics of GQDs; however, the precise effect of these approaches on PL behavior is not fully understood. Several scientists have observed that altering the surface chemistry of GQDs can alter the emission spectra of these materials; however, how these alterations change the interactions with biological tissues and imaging performance. Consequently, additional research is required to enhance the brightness, stability, and emission wavelength of GQDs as well as optimize the size, shape, and surface characteristics of GQDs to achieve the optimal emission; these efforts are necessary to improve the effectiveness of GQDs for deeper tissue imaging, in which TP and NIR emission are necessary.

For drug delivery applications, it is important to ensure that GQDs can carry drug molecules to the target location inside the body with high specificity and minimal side effects. Appropriate modification of GQDs can enable these materials to interact with specific receptors or biomarkers associated with diseases. The ability of GQDs to access biological barriers (e.g., cell membranes) is beneficial in targeted drug delivery applications. To optimize the drug delivery functionality of GQDs, further research is required to explore the mechanisms by which GQDs interact with cells and tissues. In addition, further research is necessary to explore the mechanisms by which GQDs can be engineered to improve their drug loading and release properties. Altering GQD surface properties to realize controlled drug release (e.g., delivery of the loaded drug at the desired location and time) is necessary for expanding the therapeutic effectiveness of GQD‐based drug delivery systems. The biocompatibility of GQDs must be systematically evaluated via temporal safety and in vivo toxicity studies. While preliminary reports indicate that GQDs are relatively safe, further studies are required to fully examine their biocompatibility and biodegradability over time, since their ability to remain stable and nontoxic inside the body is an important requirement for their use in clinical settings.

Despite these challenges, the future of GQDs in bioimaging and drug delivery is incredibly promising. As research efforts continue to elucidate more efficient GQD synthesis methods, GQDs are poised to play an important role in advancing both diagnostic and therapeutic applications. Their ability to be customized for specific imaging and drug delivery tasks, combined with their relatively low toxicity, makes them an ideal candidate for next‐generation medical technologies. Continued GQD research efforts should emphasize optimizing the PL properties of GQDs, enhancing the QY of GQDs, developing more efficient methods for GQD surface modification, and exploring GQD behavior in real‐time biological environments. If the abovementioned challenges are overcome, GQDs are well placed to offer a safe, effective, and versatile solution for a variety of biomedical applications.

## Author Contributions

Sachin Kadian and Shubhangi Shukla equally designed, collected the data, wrote, reviewed, and edited the original and revised manuscript draft. Amit K. Yadav, Brahamdutt Arya, Sushant Sethi, and Vishal Chaudhary designed the figures and tables and assisted in the preparation of the original and edited drafts. Roger Narayan arranged the funding, supervised, reviewed, and edited the original and revised draft. All authors read and approved the final manuscript.

## Conflicts of Interest

The authors declare no conflicts to interest.

## Ethics Statement

The authors have nothing to report.

## Data Availability

The data that support the information in this article is available from the corresponding author upon reasonable request.
